# Nutrition and immunity in newborns

**DOI:** 10.1186/1824-7288-41-S1-A33

**Published:** 2015-09-24

**Authors:** Antonietta Giannattasio, Valentina Marra, Stefania Zoccali, Letizia Capasso, Francesco Raimondi

**Affiliations:** 1 Department of Translational Medical Sciences-Section of Neonatology, University of Naples Federico II, Naples Italy

## 

Through fetal life, infancy and childhood, the immune system undergoes a process of functional maturation. The adequacy of this process is dependent on environmental factors, and there is strong evidence of the impact of pre- and post-natal nutrition in this regard [1]. The early postnatal period is a critical window for immune maturation of newborns. Exclusive human milk feeding for the first 6 months of life is recognized as the normative standard for infant feeding [2,3]. Human milk contains many hundreds to thousands of distinct bioactive molecules that protect against infection and inflammation as cytokines, nucleotides, hormones, and growth factors [2,3]. These specific protective components are so numerous, that science is just beginning to understand their functions. One of these is osteopontin. Human colostrum is very rich in osteopontin and mature human milk contains8 fold more osteopontin than cow's milk. Recent studies suggest that human milk osteopontin might regulate inducible nitric oxide synthase and synthesis of nitric oxide, improve barrier function and reduce inflammation. Thereby mediating cell attachment, migration, chemotaxis and intracellular signaling, osteopontinhas important barrier functions, protecting the intestinalmucosa against drug-induced colitis in murine models, increasing the levels ofTGF-beta1 and decreasing pro-inflammatory cytokines [4].

Nutrition is a key component also for the composition of intestinal microbiota [5]. Several studies have provided conclusive evidence of the critical role of the intestinal microbiota in regulating both mucosal and systemic immunity [5]. Immediately after delivery, the human infant acquires a much complex microbiota. It is known that intestinal microbiome composition and diversity are affected by several factors as maternal clinical conditions, route of delivery, antibiotic administration and infant diet (breast- versus formula-fed infants). In breast-fed babies, Bifidobacteria and Lactobacilli quickly become dominant, whereas in formula-fed babies, Bacteroides species are prevalent, alongside other bacteria known to be enteric pathogens. Furthermore, breast milkcontains high concentrations of oligosaccharides, which ferment in the bowel and promote the growth of Bifidobacterium gut commensals. Dysbiosis in early life has been associated with immune-mediated childhood disorders and obesity [5]. 

In the long-term, nutritional strategies might improve the development of the microbiome and intestine with the final aim to enhance the clinical care of high-risk infants as low birth weight and premature infants. However, nutritional research to date suggests that there will not be a simple or single nutritional intervention but multiple actions are needed to reverse the association between malnutrition and infection.
